# Characterization of the complete plastome of *Cyperus rotundus L.* (Cyperaceae)

**DOI:** 10.1080/23802359.2020.1845999

**Published:** 2021-01-13

**Authors:** Renjie Wu, Cong Yu, Yang Wu

**Affiliations:** a College of Life Science, Jinggangshan University, Ji’an, China; b Institute of Physical Education, Jinggangshan University, Ji’an, China

**Keywords:** *Cyperus rotundus*, chloroplast genome, Cyperaceae, phylogenetic

## Abstract

*Cyperus rotundus* L. (*C. rotundus*) is a sedge belonging to the family Cyperaceae and is widely distributed in tropical and warmer temperate regions worldwide. It is one of the oldest traditional medicinal herbs in China, India, Japan, and Korea. In this study, we sequenced the complete chloroplast genome of *C. rotundus* on the Illumina HiSeq Platform. The chloroplast genome is 182,986 bp in length, with a typical quadripartite structure and consisting of a pair of inverted repeat (IR) regions (35,969 bp) separated by a large single-copy (LSC) region (100,733 bp) and a small single-copy (SSC) region (10,315 bp). It was predicted to contain a total of 133 genes, with an overall GC content of 33.26%. Phylogenetic analysis suggested *C. rotundus* is sister to *Eleocharis celluosa* and *Eleocharis dulcis*.

*Cyperus rotundus* L. (Cyperaceae) is a medicinal herb traditionally used to treat various clinical conditions at home such as diarrhea, diabetes, pyresis, inflammation, malaria, and stomach and bowel disorders (Peerzada et al. 2015). Currently, it is one of the most widespread, problematic, and economically damaging agronomic weeds, growing wildly in various tropical and subtropical regions of the world (Qasim et al. 2014). It is a perennial, monocotyledonous and herbaceous plant of the Cyperaceae family (Himaja et al. 2014). The immense distribution of the nut-grass is due to its capacity to adapt divergent environmental conditions, altitudes, climates, moisture level and soil pH (Anand et al. 2019). Despite extensive studies on the phytochemistry, pharmacology and medicine of *C. rotundus* (Peerzada et al. 2015), the genetic information for this species remains quite limited (Okoli et al. 1997). It is often confused with other *Cyperus* species, and there are few studies on the *C. rotundus* genome so far. Here, we assembled and characterized the complete chloroplast genome sequence of *C. rotundus* to provide information for the identification of *Cyperus*, as well as assist further phylogenetic study of Cyperaceae.

In this study, the samples of *C. rotundus* were collected from Lulin ecological park, Ji’an, Jiangxi Province, China (N 27°08′ 24.01′′, E 115°0′ 33.26′′). The voucher specimen of *C. rotundus* has been kept in Key Laboratory of Ecological Environment and Resource Utilization, Jinggangshan University (accession number: JGSU20200628). The total genomic DNA was extracted from fresh leaves using DNeasy Plant Mini Kit (Qiagen, Valencia, CA). The DNA was stored at −20 °C in our lab until use. The whole-genome sequencing was conducted as the paired-end using the Illumina HiSeq 2000 platform (Illumina, San Diego, CA). Illumina paired-end sequencing generated 1,683,089 bp raw reads after adapters were removed. The raw reads were filtered by CLC Genomics Workbench v9, and the obtained clean reads were assembled into chloroplast genome using SPAdes assembler 3.10.0 (Bankevich et al. 2012). Finally, Gene annotation was performed using the CpGAVAS pipeline (Liu et al. 2012). The obtained sequence was submitted to GenBank under the accession number MT937176.

The complete chloroplast genome of *C. rotundus* has a typical quadripartite structure and is a circular molecule 182,986 bp in length, consisting of two inverted repeats (IR) regions of 35,969 bp, separated by large single-copy (LSC) and small single-copy (SSC) regions of 100,733 bp and 10,315 bp, respectively. The overall GC content of the chloroplast genome was 33.26%, whereas the corresponding values of the LSC, SSC, and IR regions were 30.91%, 25.11%, and 37.73%, respectively. A total of 133 genes were annotated in the sequenced *C. rotundus* chloroplast genome, containing 41 transfer RNAs, 8 ribosomal RNAs and 84 protein-coding genes. These genes belong to several categories with different functions, and 34 duplicated genes are located in the IR regions, including 14 protein-coding genes (rps3, rpl22, rps19, rpl2, rps7, ndhB, rpl32, ycf68, rps15, ndhH, ndhA, ndhI, ndhG, and rpl33), seven tRNA genes (trnH-GUG, trnM-CAU, trnL-CAA, trnI-GAU, trnA-UGC, trnR-ACG, and trnN-GUU), and four rRNA genes (rrn16, rrn23, rrn4.5, and rrn5). The transcription regulation of genes was believed to be affected by introns and exons. There are 100 unique genes, among which 16 genes contained one intron, and one gene (ycf3) contained two introns. Chloroplast genomes have been proven to significant in reconstructing phylogenetic relationships (Hong et al. 2017). To investigate the relationship of *C. rotundus*, the chloroplast genomes of *C. rotundus* and 8 other species from Cyperaceae were aligned using MAFFT ver. 7.307 (Katoh and Standley 2013). A phylogenetic tree (Figure 1) was constructed with the maximum likelihood method using RAxML (Stamatakis 2014). The result of the phylogenetic analysis revealed that *C. rotundus* is not monophyletic. The *C. rotundus* is sister to *Eleocharis celluosa* and *Eleocharis dulcis*. The complete plastid genome sequence of *C. rotundus* will provide genetic and genomic information to promote its horticulture, officinal utilization and systematics research of Cyperaceae.

**Figure 1.**
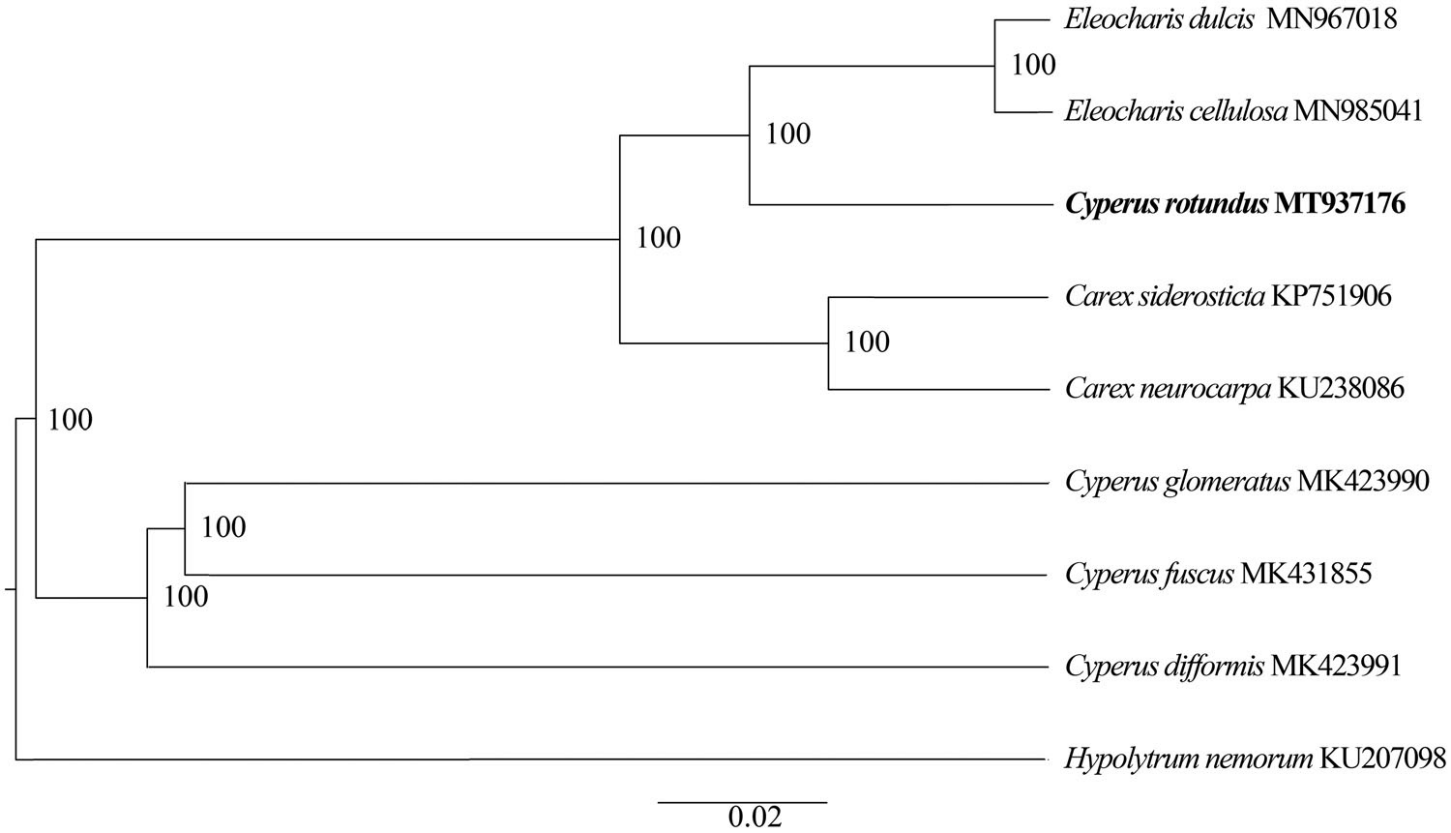
Phylogenetic tree inferred by maximum-likelihood (ML) method based on the complete chloroplast genomes of 8 representative species. Numbers near the nodes mean bootstrap support value.

## Data Availability

The data that support the findings of this study are openly available in GenBank of NCBI at https://www.ncbi.nlm.nih.gov, reference number MT937176.
